# Retraction: Celastrol inhibit the proliferation, invasion and migration of human cervical HeLa cancer cells through down‐regulation of MMP‐2 and MMP‐9

**DOI:** 10.1111/jcmm.18326

**Published:** 2024-04-29

## Abstract

Zhang J, Wang R, Cheng L, Xu H. Celastrol inhibit the proliferation, invasion and migration of human cervical HeLa cancer cells through down‐regulation of MMP‐2 and MMP‐9. *J Cell Mol Med*. 2021;25:5335‐5338. doi: 10.1111/jcmm.16488

The above article, published online on 4 May 2021 in Wiley Online Library (wileyonlinelibrary.com), has been retracted by agreement between the journal Editor‐in‐Chief, Stefan N. Constantinescu, and the Foundation for Cellular and Molecular Medicine/John Wiley & Sons Ltd. Following publication, concerns were raised by third parties that portions of Figure 1 are identical to Figure 1 presented in another article (1) from a different author group and covering a different research topic. Further investigation confirmed these concerns and revealed additional duplication in Figures 1 and 2, overlapping with Figures 1 and 3 in (1). The authors of both articles were approached for an explanation but the authors of this article did not respond. The authors of (1) were unable to provide any underlying data. The retraction has been agreed because of concerns regarding the integrity of the research presented given that the figures were duplicated.

(1) Shi X, Luo X, Chen T, et al. Naringenin inhibits migration, invasion, induces apoptosis in human lung cancer cells and arrests tumour progression in vitro. *J Cell Mol Med*. 2021;25:2563‐2571. doi: 10.1111/jcmm.16226

Zhang J, Wang R, Cheng L, Xu H. Celastrol inhibit the proliferation, invasion and migration of human cervical HeLa cancer cells through down‐regulation of MMP‐2 and MMP‐9. *J Cell Mol Med*. 2021;25:5335‐5338. doi: 10.1111/jcmm.16488

The above article, published online on 4 May 2021 in Wiley Online Library (wileyonlinelibrary.com), has been retracted by agreement between the journal Editor‐in‐Chief, Stefan N. Constantinescu, and the Foundation for Cellular and Molecular Medicine/John Wiley & Sons Ltd. Following publication, concerns were raised by third parties that portions of Figure 1 are identical to Figure 1 presented in another article (1) from a different author group and covering a different research topic. Further investigation confirmed these concerns and revealed additional duplication in Figures 1 and 2, overlapping with Figures 1 and 3 in (1). The authors of both articles were approached for an explanation but the authors of this article did not respond. The authors of (1) were unable to provide any underlying data. The retraction has been agreed because of concerns regarding the integrity of the research presented given that the figures were duplicated.

(1) Shi X, Luo X, Chen T, et al. Naringenin inhibits migration, invasion, induces apoptosis in human lung cancer cells and arrests tumour progression in vitro. *J Cell Mol Med*. 2021;25:2563‐2571. doi: 10.1111/jcmm.16226

